# Identification and Characterization of the Direct Interaction between Methotrexate (MTX) and High-Mobility Group Box 1 (HMGB1) Protein

**DOI:** 10.1371/journal.pone.0063073

**Published:** 2013-05-03

**Authors:** Yuki Kuroiwa, Yoichi Takakusagi, Tomoe Kusayanagi, Kouji Kuramochi, Takahiko Imai, Tomoko Hirayama, Ichiaki Ito, Michiteru Yoshida, Kengo Sakaguchi, Fumio Sugawara

**Affiliations:** 1 Department of Applied Biological Science, Faculty of Science and Technology, Tokyo University of Science, Yamazaki, Noda, Chiba, Japan; 2 Department of Biological Science and Technology, Faculty of Industrial Science and Technology, Tokyo University of Science, Yamazaki, Noda, Chiba, Japan; 3 National Cancer Institute, National Institutes of Health, Bethesda, Maryland, United States of America; University of Bologna & Italian Institute of Technology, Italy

## Abstract

**Background:**

Methotrexate (MTX) is an agent used in chemotherapy of tumors and autoimmune disease including rheumatoid arthritis (RA). In addition, MTX has some anti-inflammatory activity. Although dihydrofolate reductase (DHFR) is a well-known target for the anti-tumor effect of MTX, the mode of action for the anti-inflammatory activity of MTX is not fully understood.

**Methodology/Result:**

Here, we performed a screening of MTX-binding proteins using T7 phage display with a synthetic biotinylated MTX derivative. We then characterized the interactions using surface plasmon resonance (SPR) analysis and electrophoretic mobility shift assay (EMSA). Using a T7 phage display screen, we identified T7 phages that displayed part of high-mobility group box 1 (HMGB1) protein (K86-V175). Binding affinities as well as likely binding sites were characterized using genetically engineered truncated versions of HMGB1 protein (Al G1-K87, Bj: F88-K181), indicating that MTX binds to HMGB1 *via* two independent sites with a dissociation constants (K_D_) of 0.50±0.03 µM for Al and 0.24±0.01 µM for Bj. Although MTX did not inhibit the binding of HMGB1 to DNA *via* these domains, HMGB1/RAGE association was impeded in the presence of MTX. These data suggested that binding of MTX to part of the RAGE-binding region (K149-V175) in HMGB1 might be significant for the anti-inflammatory effect of MTX. Indeed, in murine macrophage-like cells (RAW 264.7), TNF-α release and mitogenic activity elicited by specific RAGE stimulation with a truncated monomeric HMGB1 were inhibited in the presence of MTX.

**Conclusions/Significance:**

These data demonstrate that HMGB1 is a direct binding protein of MTX. Moreover, binding of MTX to RAGE-binding region in HMGB1 inhibited the HMGB1/RAGE interaction at the molecular and cellular levels. These data might explain the molecular basis underlying the mechanism of action for the anti-inflammatory effect of MTX.

## Introduction

Methotrexate (MTX) is a folic acid antagonist that was conventionally developed as a clinical chemotherapeutic agent for malignancies such as leukemia [1]. The inhibition of dihydrofolate reductase (DHFR) by MTX blocks nucleotide biosynthesis, thereby retarding the proliferation of cancer cells [2]. Previously, deoxycytidine kinase (dCK), a salvage pathway enzyme of nucleotide biosynthesis, was reported as an alternative molecular target for MTX by affinity purification using styrene glycinemethacrylate (SG) beads from cytoplasmic extracts of the human acute monocytic leukemia cell line THP-1 [3].

A low-dose of MTX is currently used for the clinical treatment of inflammatory diseases, including rheumatoid arthritis (RA), due to its beneficial anti-inflammatory and immunosuppressive activities [1]. Indeed, treatment of MTX suppresses both antibody production and chemoattraction of neutrophils at the diseased area of tissue as well as the proliferation of lymphoma cells, blood endothelial cells and synovial fibroblasts. Suppression of nuclear factor (NF)-κB activation is likely to be involved in the mechanism of action of MTX [4]. However, because these diseases can cause chronic inflammation of unknown etiology [1], the precise way in which MTX operates in terms of anti-inflammatory activity is not fully understood. Thus, the identification of MTX binding proteins will greatly assist in determining the mode of action of MTX as well as elucidating the molecular basis of inflammatory diseases.

In order to reveal direct binding proteins of MTX, we employed the T7 phage display system [5]. The principle of the technology is based on fusing nucleotide sequences of random polypeptides to that of a phage coat protein, which enables the display of the chimeric protein on the phage surface. This characteristic allows facile determination of the amino-acid sequence by simply sequencing the DNA of the corresponding phage. By selection with the substrate of interest immobilized on a solid support as bait, phages selectively recognizing the substrate are subsequently enriched by repeated rounds of biopanning (interaction, washing, elution and amplification). Unlike filamentous phage-based systems, T7 phage facilitates a comprehensive screen for binding proteins that relies on the primary amino acid sequence of up to 1200 of amino acids, even if the target is extracellularly-secreted cytokines or growth factors with rapid turnover or low abundance [5]. Our screening of MTX-binding proteins identified part of high mobility group box 1 protein (HMGB1) as a potential direct binding region of MTX. HMGB1 is a non-histone nuclear protein that acts as an architectural chromatin-binding factor in nuclei as well as an extracellular mediator of inflammation in the form of a multifunctional alarmin driving autoimmune and inflammatory disease [6], [7], [8]. By employing surface plasmon resonance (SPR) analysis, electrophoretic mobility shift assay (EMSA) and a cell-based assay, we demonstrated that binding of MTX to HMGB1 through part of the receptor for advanced glycation end products (RAGE)-binding region (K149-V175) interfered with HMGB1/RAGE interaction, thereby inhibiting HMGB1-elicited TNF-α release and mitogenic activity [9]. These data might explain the molecular basis underlying the mode of action for the anti-inflammatory effect of MTX, which may provide guidance for further experimentation and clinical treatment of inflammatory diseases, including RA.

## Materials and Methods

### Synthesis of bio-MTX

**Figure 1:**
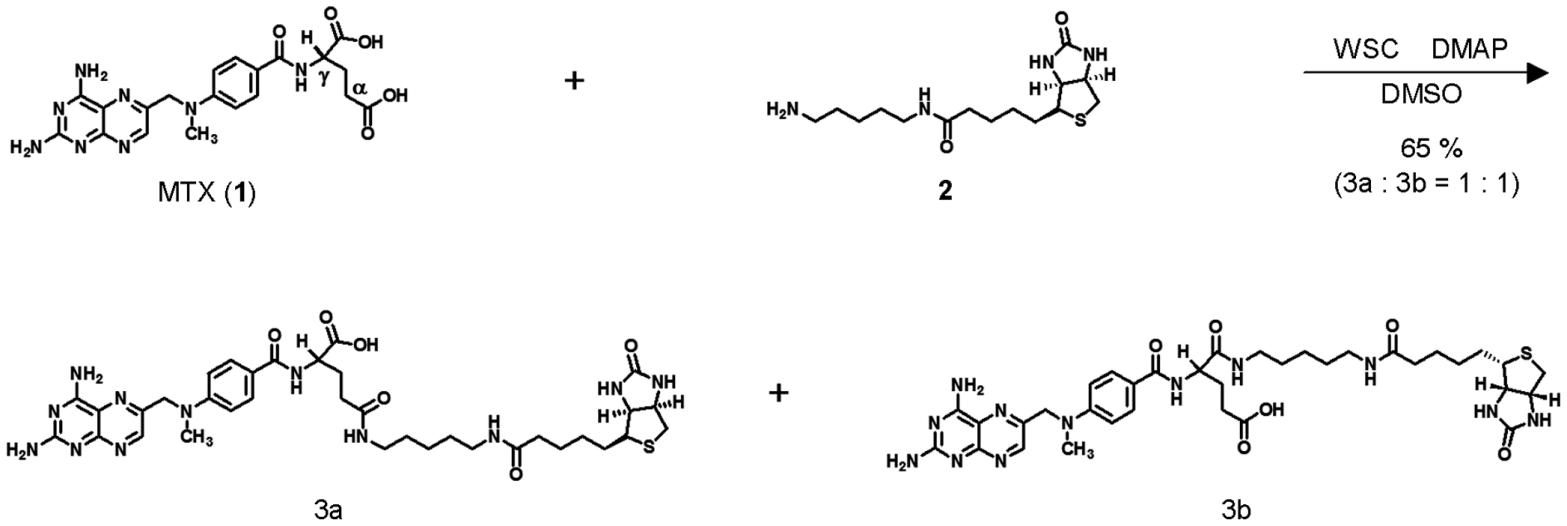
Synthesis of bio-MTX (3). The reaction gave bio-MTX **3a** and **3b** as a 1∶1 mixture.

A biotinylated derivative of MTX (bio-MTX) was synthesized according to previous reports (Figure 1) [10], [11]. MTX (**1**) (10.0 mg, 0.022 mmol) and 5-(biotinamido) pentylamine (**2**) (10.8 mg, 0.033 mmol) in DMSO (0.4 mL) was added to a solution of WSC (4.5 mg, 0.037 mmol) followed by DMAP (3.4 mg, 0.022 mmol). The mixture was then stirred overnight at room temperature. The resulting solution was concentrated *in vacuo* and the reaction products purified by reverse phase column chromatography (Wakogel® 100C18, Wako Pure Chemical Industries, Ltd., Osaka, Japan) with 0.1 M NH_4_HCO_3_ aq. and MeOH to give a mixture of bio-MTX (**3a**, **3b**) in about 65% yield as a 1∶1 ratio.^ 1^H NMR spectra were recorded on a Bruker 600 MHz spectrometer (Avance DRX-600) using a mixture of DMSO-*d*_6_ and D_2_O as a solvent. Chemical shifts are expressed as δ (ppm) relative to residual solvent resonance (CHD_2_SOCD_3_), and coupling constants (*J*) are expressed in Hertz. Mass spectra (MS) were obtained on an Applied Biosystems mass spectrometer (APIQSTAR pulsar i) under high resolution conditions using (poly)ethylene glycol as an internal standard.

^1^H NMR (600 MHz, DMSO-D_2_O): δ 8.56 (1H×2, s), 7.60 (2H, d, *J* = 8.8 Hz), 7.58 (2H, d, *J* = 8.9 Hz), 6.75 (2H, d, *J* = 8.9 Hz), 6.74 (2H, d, *J* = 8.8 Hz), 4.72 (2H×2, s), 4.43–4.36 (1H×2, m), 4.35 (1H, *br*t, *J = *5.8 Hz), 4.32 (1H, *br*t, *J = *5.5 Hz), 4.12 (1H×2, m), 3.13 (3H, s), 3.13 (3H, s), 3.08 (1H×2, m), 3.03–2.91 (4H×2, m), 2.77 (1H, dd, *J* = 12.6 Hz, 4.5 Hz), 2.73 (1H, dd, *J* = 12.6 Hz, 4.9 Hz), 2.55 (1H, d, *J* = 12.6 Hz), 2.52 (1H, d, *J* = 12.6 Hz), 2.19–1.91 (2H×2, m), 2.03 (2H, t, *J = *7.0 Hz), 1.99 (2H, t, *J = *7.1 Hz), 1.80 (2H×2, m), 1.57–1.10 (12H×2, m). HRMS (ESI): calcd for C_35_ H_48_N_12_O_6_SNa ([M+Na]^+^) *m/z* 787.3432, found *m/z* 787.3423.

### Construction of the T7 Phage Library

The T7 phage library was constructed using the T7Select10-3® OrientExpress cDNA cloning System, Random Primer (Novagen, Darmstadt, Germany) and T7Select10-3® Cloning Kit (Novagen) according to the manufacturer’s instructions [12]. A cDNA library, constructed from mRNA derived from MH14 hepatoma cells, was kindly provided by Dr. Sahara (Sapporo Medical University, Sapporo, Japan). The primary titer of this library was 4.8×10^5^ pfu/ml. For the biopanning, the phage library was amplified up to 10^10^ pfu/ml using *E. coli* (BLT5615) as the host strain.

### Screening of a Library of T7 Phage-displayed Polypeptide

A modified version of the panning procedure given in the manufacturer’s instructions (Novagen) was used in this study [13]. Bio-MTX (100 pmol) was immobilized onto a streptavidin (SA)-coated 96 well plate (Nalge Nunc International, Wiesbaden, Germany) after dilution in 200 µL of TBS buffer (50 mM Tris-HCl, 150 mM NaCl, pH 7.4). The solution was incubated for 1 h at room temperature. After washing each well in TBD buffer, 200 µL of TBS buffer containing 3% skimmed milk was added and left for 1 h at room temperature for blocking. The blocking solution was then discarded and 100 µL of T7 phage library or amplified phage solution (approximately 10^9^ pfu/well) was added. After 1 h incubation at room temperature, the phage solution was removed and each well was washed 10 times with 200 µL of TBSDT buffer (50 mM Tris-HCl, 150 mM NaCl, 10% DMSO, 0.1% Tween 20, pH 7.4). The remaining phage particles were dissociated using 100 µL of TBSDT buffer containing 1% SDS for 1 h. The titer of phage was measured and then incubated immediately with 2 ml of host *E. coli* (BLT5615) with shaking at 37°C until lysis was observed. The phage solution was amplified up to approximately 10^10^ pfu/ml and then subjected to the next round of biopanning. As a control, MTX-non-immobilized well was prepared and tested according to the same procedure.

### Sequencing of T7 Phage DNA

Phage particles were isolated from the eluted solution after the fifth round of screening and then sequenced. The sequencing was achieved by first amplifying the cDNA insert of each phage using the following pair of oligonucleotide primers: forward primer 5′-TGCTAACTTCCAAGCGGACC-3′; reverse primer 5′-TTGCCCAGAACTCCCCAA-3′. The PCR products were purified and treated with BigDye Terminator v3.1 Cycle Sequencing Kit (Applied BioSystems, Foster City, CA). Sequencing was performed on an ABI PRISM® 3100 Genetic Analyzer (Applied BioSystems) and the results were analyzed using BLAST (http://www.ncbi.nlm.nih.gov/blast/Blast.cgi).

### Affinity check of monoclonal T7 phage by competition with MTX

The affinity between immobilized MTX and either HMG-displaying monoclonal phage or control phage (1.0×10^8^ pfu/ml) were determined using the same protocol as described for the biopanning. An aliquot 100 µl of each phage solution was employed for the test. TBSDT with or without 200 µM MTX was used as an elution buffer [14].

### Preparation of Recombinant Protein

**Figure 2:**
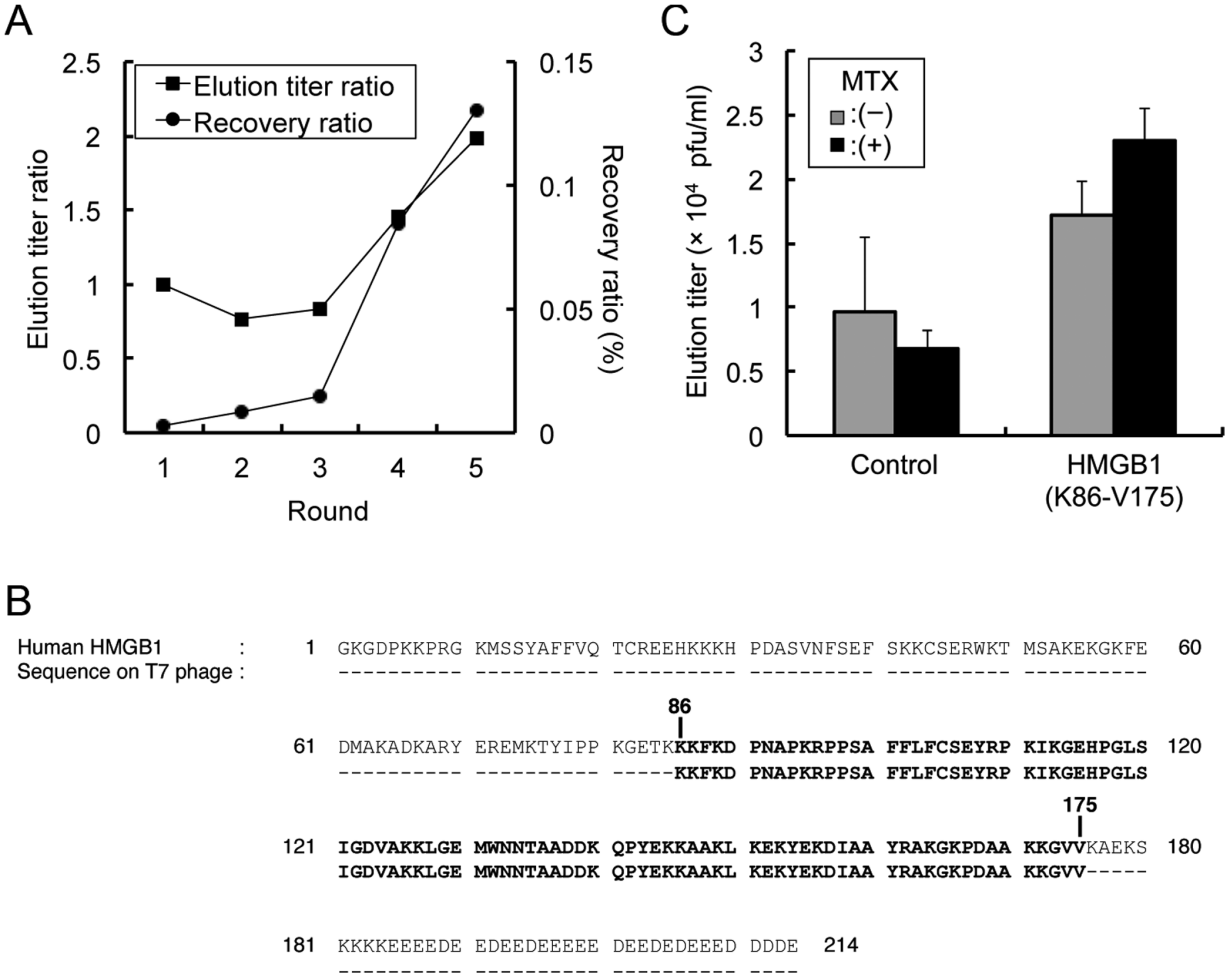
Identification of MTX-binding protein by T7 phage display. (A) Elution titer and recovery ratio after each round of biopanning. Elution titer ratio = Number of eluted phage particles (pfu/ml) from test well/Number of eluted phage particles (pfu/ml) from the MTX-non-immobilized control well. Recovery ratio (%) = [Number of the eluted phage particles (pfu/ml)/Number of input phage particles (pfu/ml)]×100. Pfu: plaque forming unit. (B) Sequence homology between human HMGB1 and the coding polypeptide of MTX-binding T7 phage particle. The recovered sequence encodes a polypeptide that is 100% identical to a portion of human HMGB1 (K86-V175). (C) The specific affinity of HMGB1-displaying T7 phage to MTX. Isolated HMGB1 (K86-V175)-displaying monoclonal T7 phage or control T7 phage (no cDNA insert) stocks were individually allowed to interact with immobilized MTX. Bound phages were then eluted using buffer containing an excess (200 µM) of MTX [MTX (+)] or no MTX [MTX (−)]. The number of eluted phage particles was counted. N = 3; data represented as mean ± SE.

Bj (amino acid residues F88-K181 of HMGB1), Al (G1-K87) and AlBj (G1-K181) proteins (Figure 2A) were individually engineered for heterologous expression in *E. coli* JM109 using pTrcHis vector (Invitrogen, Carlsbad, CA). In each case, the corresponding recombinant protein included an N-terminal His tag (6×His residues) [15]. The transformant was grown in LB medium containing carbenicillin at 37°C and heterologous gene expression was induced with 0.5 mM isopropyl-β-**D**-thiogalactopyranoside (IPTG). The cell culture was continued for 3 h before harvesting by centrifugation. Each cell pellet was suspended in purification buffer (20 mM Tris-HCl, 500 mM NaCl, 10 mM 2-mercaptoethanol, pH 7.5) containing 1 mM phenylmethylsulfonyl fluoride (PMSF) and 1 mg/ml of lysozyme. Cells were disrupted by sonication and the cell-free extract was then clarified by centrifugation at 20400 *g*. The soluble fraction was filtered and loaded onto a HisTrap HP column (1 ml) (GE Healthcare, Amersham, UK) equilibrated in purification buffer containing 50 mM imidazole using a FPLC system (ÄKTA explorer, GE Healthcare). After washing the column, bound protein was eluted using a gradient of 50 mM (A) to 250 mM imidazole (B). The eluted recombinant protein was then desalted using a PD10 column (GE Healthcare). The purity was analyzed by SDS-PAGE using 15% separation gel. The bands were stained with CBB.

### Surface Plasmon Resonance (SPR) Analysis

Biacore® 3000 (GE Healthcare UK Ltd.) was used for the interaction analysis [16]. The bio-MTX was immobilized on a SA sensor chip (GE Healthcare) as a ligand. The bio-MTX (1 mM) was injected at a flow rate of 10 µl/min until 50 resonance units (RU) of MTX-biotin was immobilized. Various concentrations of each protein (Al: 0.31–5 µM, Bj: 0.16–2.5 µM) in PBS were then injected over the MTX-immobilized sensor chip at a flow rate of 30 µl/min.

For Bj-RAGE interaction analysis, extracellular domain of RAGE (M1-A344) (R&D system Inc., purity >90%) was immobilized on a CM5 sensor chip by an amine coupling reaction up to 5000 RU. Various concentrations (0.31–10 µM) of Bj protein with or without 1 mM of MTX was then injected over the RAGE-immobilized sensor chip at a flow rate of 30 µl/min.

Each response curve was generated by subtraction of the background signal generated simultaneously on the control flow cell. At least 3 independent experiments were performed for each protein. BIAevaluation 4.1 software (GE Healthcare) was used for generating global fitting curves.

### Kinetic Analysis on SPR

The saturation binding kinetics of MTX and protein were determined using i) Langmuir-plot, ii) Scatchard-plot [17] or iii) Hill-plot [18] represented by following equations:
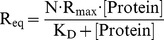
(i)

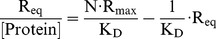
(ii)

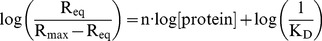
(iii)where [Protein] is the concentration of injected protein; R_eq_ is the apparent response (RU) at equilibrium obtained from SPR analysis between protein and immobilized bio-MTX on a SA sensor chip, and is obtained from curve fitting using BIAevaluation 4.1 software (GE Healthcare); N is the number of binding site per protein; R_max_ is the maximum response (RU); K_D_ is the dissociation constant between protein and immobilized bio-MTX; n is the Hill coefficients.

The binding kinetics parameter was calculated from the time-dependence of response increases. Binding between protein and bio-MTX was determined by following equation:
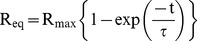
(iv)

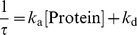
(v)where *k*_a_ is the association rate constant between Protein and bio-MTX; *k*_d_ is the dissociation rate constant; R_max_ is the maximum response (RU); τ is the relaxation time, obtained from non-linear curve fitting using BIAevaluation 3.2 software.

The effect of MTX on Bj-RAGE interaction was elucidated by vi) Langmuir-plot and vii) Lineweaver-Burk plot (Double-reciprocal plot) for a noncompetitive inhibition model represented by the following equation:
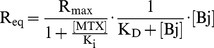
(vi)

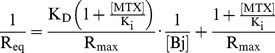
(vii)where [Bj] and [MTX] are the concentrations of each substrate; R_eq_ is the apparent response (RU) obtained from SPR analysis between Bj (F88-K181) protein and immobilized RAGE on a CM5 sensor chip; R_max_ is the maximum response (RU); K_D_ is the dissociation constant between Bj protein and immobilized RAGE; K_I_ is the dissociation constant between MTX and immobilized RAGE.

### Electrophoretic Mobility Shift Assay (EMSA)

Linear double-stranded DNA was prepared from Form III pGEM vector (Promega, Madison, WI) by digestion with *Eco*RI. Various molar ratios of AlBj (G1-K181) protein (AlBj/DNA: 0–800) were mixed with 0.5 ng of DNA with or without MTX (1 mM) in reaction buffer (10 mM Tris-HCl, pH 7.8, 100 mM NaCl, 1 mM EDTA, 10 mM MgCl_2_, l mM dithiothreitol, 10% glycerol, 40 µg bovine serum albumin) (20 µl) at 25°C for 1 h. The resulting solution was subjected to 1.0% agarose gel electrophoresis in 40 mM Tris-acetate containing 1 mM EDTA. After electrophoresis, the gel was stained with ethidium bromide.

### Cell Culture

Murine macrophage-like RAW 264.7 cells were cultured in RPMI 1640 medium (Nacalai tesque, Kyoto, Japan) supplemented with 10% heat inactivated fetal bovine serum (Japan Bioserum Co. Ltd., Nagoya, Japan) and antibiotic-antimycotic mixed stock solution (Nacalai tesque) in a humid atmosphere containing 5% CO_2_.

### TNF-α Release Measurements

RAW 264.7 cells (90% confluent), grown in a 24-well culture dish, were treated with serum-free Opti-MEM I medium (Invitrogen). The cells were then stimulated with Bj protein (0–0.5 µM) and/or MTX (0–100 µM) for 6 h. TNF-α release into the conditioned medium was then measured using an ELISA kit (Quantikine® Immunoassay Mouse TNF-α) according to the manufacturer’s instructions (R&D System Inc., Minneapolis, MN).

### Cell Proliferation Assay

RAW 264.7 cells in 96-well culture dishes (5.0×10^3^ cells/well) were cultured for 48 h and then stimulated with Bj protein (0–0.5 µM) and/or MTX (0–100 µM). The number of living cells was counted using cell count reagent SF (Nacalai tesque) by measurement of the absorbance at OD 490 nm.

### Docking Simulation

Molecular modeling between MTX and HMGB1 (K81–K164) was performed using a three-dimensional structure (PDB code: 2gzk). The final docking structure was constructed based on Discovery Studio (DS) 1.7 (Accelrys, Inc., San Diego, CA) with CDOCKER program on a PC terminal (Express, NEC) linked with Regatta (96 nodes, IBM). The binding energies of the two proteins were acquired using a solvation model and calculated by the GBSW parameter.

## Results

### Identification of MTX Binding to HMGB1 by a T7 Phage Display Screen

A biotinylated derivative of MTX (bio-MTX) was synthesized according to previous reports (Figure 1) [10], [11]. Coupling MTX (**1**) and 5-(biotinamido)pentylamine (**2**) in the presence of water soluble carbodiimide (WSC) and *N*,*N*-dimethyl-4-aminopyridine (DMAP) afforded an inseparable mixture of bio-MTX conjugates (**3a** and **3b**) as a 1∶ 1 mixture. Immobilization of MTX blocks either the α or γ carboxyl group, which may interfere with the binding of MTX to its target protein. Nonetheless, use of the mixture of both derivatives can compensate for the loss of one carboxyl group by the other form.

Using this biotinylated derivative mixture (bio-MTX) as bait, we conducted a T7 phage display screen. A library of T7 phage that displays a diversity of human proteins was employed for the affinity selection. Bio-MTX was immobilized on a streptavidin (SA)-coated 96-well plate and then incubated with the T7 phage library. After washing to remove the unbound T7 phage particles, the bound T7 phage particles were eluted with 1% SDS solution. The eluted phage particles were amplified using host *Escherichia coli* (BLT5615), and the resulting phage stock was used in the next round of biopanning. T7 phage particles displaying affinity for MTX were then efficiently enriched by repetitive rounds of biopanning. There was no significant change in the elution titer ratio and recovery ratio during the first three rounds of screening. However, these parameters markedly increased in the following two rounds, indicating an enrichment of MTX-binding T7 phage particles (Figure 2A). We randomly selected 36 individual T7 phage clones from the eluted phage stock after the fifth round of screening and subjected them to sequence analysis. One of the duplicated clones from the 36 selected T7 phage particles displayed 100% sequence match to human HMGB1 (K86-V175), identified by a homology search using BLAST (Figure 2B).

To confirm the specific affinity of MTX for HMGB1, a monoclonal T7 phage displaying a part of HMGB1 (K86-V175) was applied to a bio-MTX-immobilized well. Bound T7 phage particles were then eluted using a buffer containing an excess (200 µM) of MTX [MTX (+)] or MTX-free buffer [MTX (−)]. The phage titer in the eluate was then compared to that of control phage (no insertion of cDNA). As shown in Figure 2C, the titer of HMGB1-displaying T7 phage eluted using MTX (+) was slightly greater compared with that using MTX (−). By contrast, there was no difference in the titer of control T7 phage using MTX (+) and MTX (−) buffer. These results suggest that HMGB1 displayed on the T7 phage capsid has a specific affinity for MTX.

Given the known affinity of DHFR and dCK for MTX [2], [3], one might have anticipated isolating T7 phage particles displaying these proteins during the biopanning procedure. However, no such T7 phages were detected. This was probably due to the absence of a T7 phage clone encoding full length DHFR or dCK in the T7 phage library that was constructed from MH14 hepatoma cells. Problems associated with protein-display or insolubility most likely arise from incorrect protein folding on the capsid during T7 phage assembly during the amplification process. Alternatively, MTX immobilized by a short biotin linker may be inaccessible to the MTX-binding site in the DHFR and dCK targets.

### Elucidation of the Binding Affinity between MTX and HMGB1 by SPR Analysis

**Figure 3:**
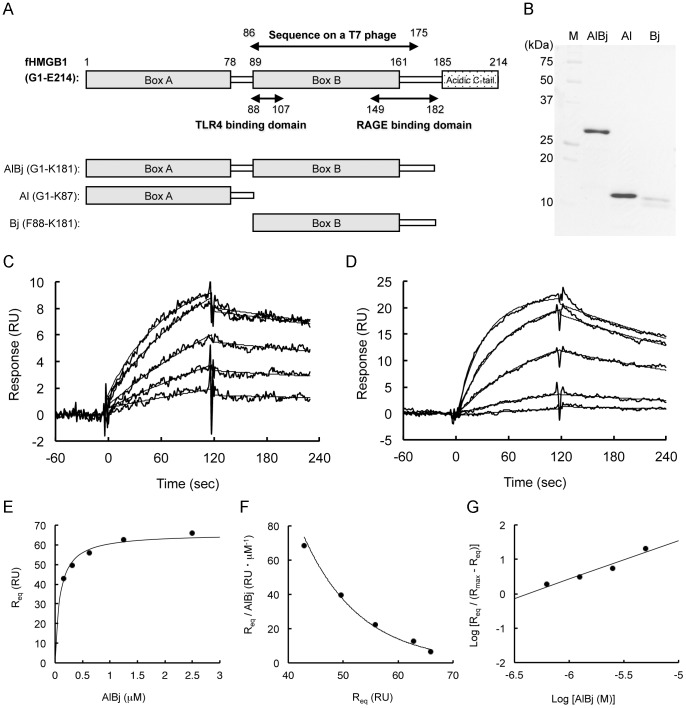
SPR analysis of the interaction between MTX and HMGB1. (A) Full length map of HMGB1 and truncated recombinant versions of the protein engineered in *E. coli*. The displayed sequence of the MTX-binding T7 phage particle (K86-V175) includes the Box B domain (K89-Y161), TLR4-binding domain (F88-E107) and part of the RAGE-binding domain (K149-V175). (B) SDS-PAGE of AlBj, Al and Bj proteins after purification by affinity chromatography. The bands were stained with CBB. (C, D) Representative SPR sensorgram with a global fitting curve between bio-MTX and Al (C) or Bj (D). Solutions containing various concentrations of Al (0.31–5 µM) or Bj protein (0.16–2.5 µM) were injected over the immobilized MTX-biotin on a SA sensor chip for 120 s and then dissociation was monitored for a further 120 s at a flow rate of 30 µl/min. Response curves were generated by subtraction of the background signals generated simultaneously on the control flow cell (bio-MTX-non-immobilized cell) and the injection of vehicle (0 µM analyte). (E) Concentration-response curve between bio-MTX and AlBj obtained from SPR analysis. R_max_ = 66. (F) Scatchard-plot analysis of AlBj binding to MTX. (G) Hill-plot analysis of AlBj binding. Hill coefficients n = 1.1. RU: resonance unit. 1 RU = 1 pg/mm^2^.

To further demonstrate the specific affinity of MTX for HMGB1 and characterize the affinity status, real-time binding of MTX to truncated HMGB1 proteins was monitored *in vitro* by surface plasmon resonance (SPR) analysis [16]. After immobilizing biotinylated MTX (bio-MTX) on the surface of a sensor chip SA (GE Healthcare), various concentrations of truncated histidine-tagged segments of HMGB1 (Figure 3A), purified by an affinity chromatography procedure as a single band on SDS-PAGE with CBB staining (Figure 3B), were injected over the immobilized bio-MTX to measure the respective binding affinities.

Figure 3C and D show representative sensorgrams with global fitting curves (BIAevaluation 4.1) obtained by the injection of various concentrations of Al (G1-K87) (0.31–5 µM) or Bj (F88-K181) (0.16–2.5 µM) protein, which contain Box A (G1-I78) or Box B (K89-Y161), respectively (also refer to Figure S1, S2) [19], [20]. Although T7 phage display identified a polypeptide corresponding to K86-V175 in Bj that contains Box B domain, Al protein also responded to the immobilized bio-MTX. Kinetic analysis showed the dissociation constant (*K*_D_) between immobilized MTX and Al or Bj to be 0.50±0.03 µM and 0.24±0.01 µM, respectively (assuming a simple 1∶1 association with the respective binding site) (Tables 1, S1 and S2). SPR analysis was also performed in which AlBj protein was injected onto immobilized MTX. Figure 3E shows a concentration-response curve, which was obtained from the global fitting of the association process on SPR sensorgrams (also refer to Figure S3 and Table S3). If AlBj associates with MTX in a 1∶1 stoichiometry (N = 1 in Scatchard-plot of equations ii)), or a 1∶2 stoichiometry (N = 2) with identical affinity strength at both sites, this plot will be a straight line [17]. However, as shown in Figure 3F, the plot showed nonlinearity. This finding indicates that there may be more than two MTX-binding sites in AlBj and the respective affinity strength with MTX at the two sites may be different. The interaction between AlBj and MTX was further analyzed using a logarithmic Hill plot of equations iii) [18]. By fitting the raw data in this model, the value of the Hill coefficient (n) can be obtained from the slope. This parameter describes the cooperativity of ligand binding. In the case of cooperative binding, once one ligand molecule is bound to the macromolecule the affinity for the second ligand molecules may increase or decrease i.e., binding is positively or negatively cooperative, showing n>1 or n<1, respectively. Alternatively, if the affinity of the second ligand is unaffected by the presence of the first ligand, the binding is noncooperative and the Hill coefficient n will be 1. Our analysis of the HMGB1/MTX interaction gave a Hill coefficient of 1.1, suggesting that binding of one MTX molecule to HMGB1 has little effect on the binding affinity of the second MTX molecule. Taken together, our results suggest that MTX is a bivalent ligand for HMGB1, which binds to the Al and Bj regions with different affinity strength.

**Table 1:** Kinetic parameters of each ligand-analyte interaction.

Ligand	Analyte	*k*_a_ (×10^3^ M^−1^s^−1^)	*k*_d_ (×10^−3^s^−1^)	K_D_(*k*_d_/*k*_a_) (µM)	R_max_ (RU)
bio-MTX	Al	8.66±5.43	4.55±2.98	0.50±0.03	9.1±3.4
bio-MTX	Bj	12.6±0.26	3.06±0.02	0.24±0.01	16.0±3.2
RAGE	Bj	0.15±0.01	0.52±0.25	3.71±1.68	268.8±46.5
RAGE	MTX	–	–	>100	–

Analytical conditions: PBS, 25°C. *k*_a_: association rate constant, *k*_d_: dissociation rate constant, K_D_: dissociation constant, R_max_: maximum binding amount. RU: resonance unit. 1 RU = 1 pg/mm^2^.

### MTX Inhibits Binding of HMGB1 to RAGE but not to DNA

**Figure 4:**
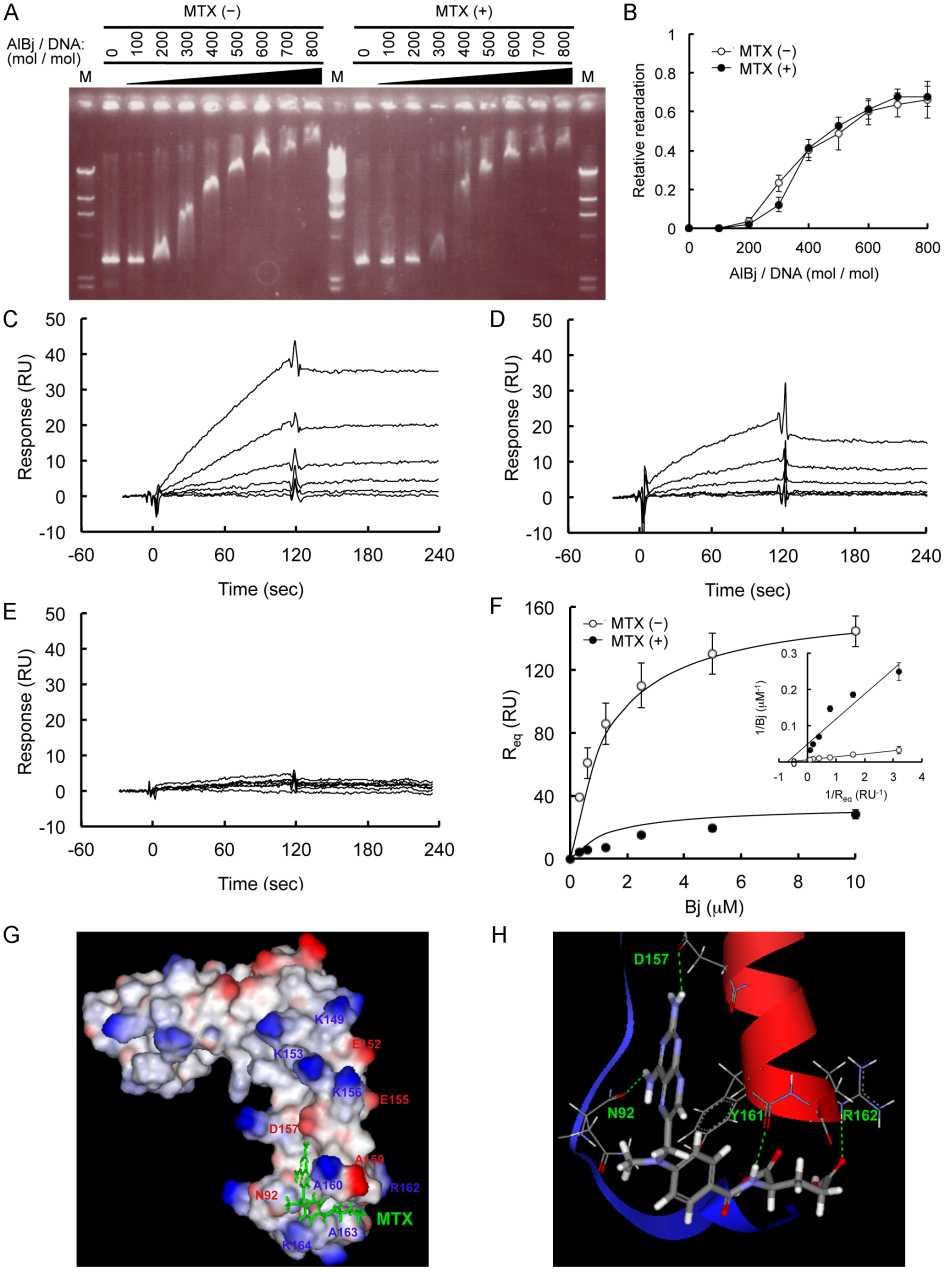
Effect of MTX for the HMGB1 binding to DNA or to RAGE. (A) Electrophoretic mobility shift assay (EMSA). The linearized form III pGEM DNA vector (0.5 ng) and AlBj protein was complexed for 1 h at various molar ratios of AlBj/DNA (0–800) in the absence (−) or presence (+) of MTX (1 mM). (B) The relative retarding distance of the DNA band was plotted against the molar ratio (0–800) of AlBj protein to DNA. Relative retardation = Distance from applied well to DNA band/Distance from applied well to control DNA band. N = 3; data represented as mean ± SE. (C–E) Representative SPR sensorgram with global fitting curve between Bj protein and RAGE (purity >90%) immobilized on a CM5 sensor chip. Six or seven different concentrations of Bj protein were injected over the immobilized RAGE for 120 s and then dissociation was monitored for a further 120 s at 30 µl/min. (C, D) Interaction between Bj protein (0.31–10 µM) with immobilized RAGE in the absence (C) or presence (D) of MTX (1 mM). (E) Interaction between MTX (1–63 µM) with immobilized RAGE. (F) Concentration-response curve and Lineweaver-Burk plot between Bj protein and RAGE in the absence (open circle) or presence (filled circle) of MTX (1 mM). Linear equations in Lineweaver-Burk plot are as follows; [MTX (−)]: y = 0.0083x +0.0061 (r^2^ = 1), K_D_ = 1.35 µM, R_max_ = 163, [MTX (+)]: y = 0.0686x +0.0493 (r^2^ = 0.90), K_D_ = 1.39 µM, R_max_ = 33, K_I_ = 142 µM. (G, H) Computer-aided binding model between MTX and HMGB1. (G) Molecular modeling of the MTX binding site in HMGB1 (K81–K164) potentially involved in the interference of HMGB1/RAGE interaction was derived using the NMR structure of fragments associated with previously published data (PDB accession code 2gzk). The predicted binding structure was solved using DS 1.7 with the CDOCKER application program by calculating the binding energy in an aqueous environment. (H) Expansion diagram of the MTX-binding site within part of RAGE-binding region (K149-V175). N92, D157, Y161 and R162 form hydrogen bonds with MTX. The binding energy is −29.31 kcal/mol.

The peptide sequence identified by our T7 phage display screen (K86-V175) overlaps three significant regions for inflammatory activities of HMGB1. One is Box B (K89-Y161) for DNA recognition to enhance intranuclear transcription of cytokines [21], [22], [23]. Second is F88-E107 in Box B, which constitutes the minimal epitope for inducing TNF-α release *via* TLR4 binding [24], [25], and the third is K149-K175, which plays an important role in binding to RAGE, a specific receptor for HMGB1 on the cell-surface that can facilitate many extracellular events (Figure 3A) [9], [26]. To clarify whether binding of MTX to HMGB1 inhibits the biological events *via* these regions, we initially performed EMSA [15], [27]. Previously, we reported that AlBj protein, which contains two DNA binding domains (Box A and B) [19], [20], binds to DNA with a dissociation constant (K_D_) of 0.95±0.37 µM as determined by SPR analysis [15]. However, the binding of AlBj protein to DNA was not inhibited in the presence of MTX at a concentration of 1 mM (Figure 4A, B), despite the stronger affinity of MTX for Box A and B domains over that of AlBj for DNA (Table 1).

Next, we investigated whether MTX affects the interaction between recombinant HMGB1 and chimeric receptors on the cell surface. The T7 phage display suggested that posttranscriptional modification or complex formation of HMGB1, which is essential for binding of HMGB1 to cognate receptors such as TLR or IL-R [28], is irrelevant for MTX/HMGB1 interaction because posttranscriptional modifications are absent in T7 phage-displayed chimeric proteins. Furthermore, although HMGB1 assembles with LPS and induces conformational changes of LPS to enhance the LPS-TLR4 signaling *via* F88-E107 (Figure 3A) [28], [29], [30], MTX does not inhibit the LPS+IFNγ-elicited TNF-α release mediated by TLR4 [31]. Based on these considerations, we hypothesized that MTX may bind to part of the RAGE-binding region (K149-V175) and show the inhibitory effect of HMGB1-RAGE interaction in which modification or complex formation of HMGB1 is unnecessary. Figure 4C shows a representative SPR sensorgram in which global fitting has been performed using BIAevaluation 4.1 software (also refer to Figure S4). Bj protein (Figure 3B) at various concentrations was injected onto chimeric RAGE (purity >90%) immobilized onto a CM5 sensor chip by an amine coupling reaction. The SPR response increased in proportion to the Bj protein concentration. From a kinetic analysis, the dissociation constant of this interaction was determined to be 3.71±1.68 µM (Table 1), agreeing with the fact that monomeric HMGB1 can directly interact with RAGE. As shown in Figures 4D and S5, this interaction was markedly weakened in the presence of MTX. No direct interaction of MTX with RAGE could be observed up to 63 µM of MTX (Figure 4E). Furthermore, the equilibrium (R_eq_) plot and subsequent Lineweaver-Burk plot revealed that MTX inhibits RAGE non-competitively (Figure 4F, Table S4), supporting the data that suggests MTX binds directly to Bj (Figure 3D) but not to RAGE (Figure 4E), and inhibits the HMGB1/RAGE interaction.

Thus, our results demonstrate that MTX does not inhibit DNA binding it does inhibit the HMGB1/RAGE interaction by binding to HMGB1 [9], [26]. This finding contrasts with glycyrrhizin, a bivalent HMGB1 ligand that binds to the shallow concave surface in Box A (G1-I78) and B (K89-Y161) in HMGB1 and moderately inhibits DNA binding [32]. Our T7 phage display screen identified a K86-V175 fragment, suggesting the K149-V175 region in the RAGE binding domain may be the “hot spot” for binding (Figure 3A). To simulate binding of MTX to this potential hot spot, a three-dimensional structure of the MTX molecule and HMGB1 (K81–K164, PDB code: 2gzk) was constructed using energy minimization. Several initial structures of HMGB1 bound to MTX were manually generated with the hot spot and then optimized using the Discovery Studio (DS) 1.7 (Accelrys, Inc.) program. In the resulting model (Figure 4G, H), MTX established favorable hydrogen bonding with N92, D157, Y161 and R162 that comprise part of an alpha helix (helix III) in the hot spot region, which stabilized the binding energy to as low as –29.31 kcal/mol.

### MTX Inhibits HMGB1-elicited TNF-α Release and Mitogenic Activity in RAW 264.7 Cells

**Figure 5:**
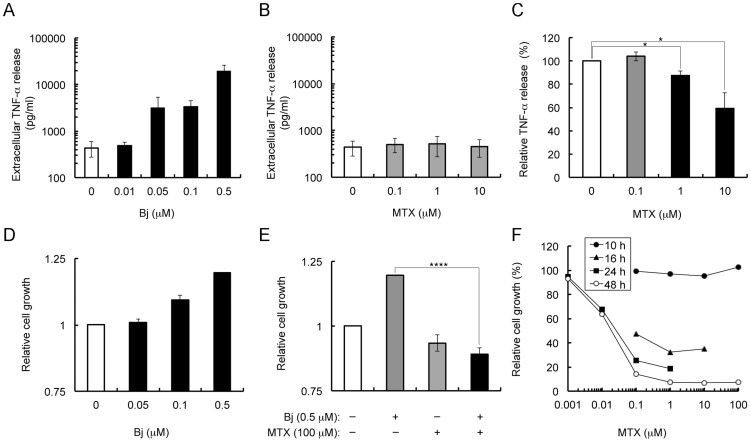
Effect of MTX on the truncated HMGB1 (Bj)-elicited TNF-α release and mitogenic activity in RAW 264.7 cells. (A) Bj protein-dependent TNF-α release. RAW 264.7 cells, in a 24-well culture dish format, were stimulated with the indicated concentrations of Bj protein for 6 h. The amount of TNF-α released into the conditioned medium was then determined by ELISA. N ≥2. (B) Influence of MTX alone for TNF-α release. RAW 264.7 cells were stimulated with 0–10 µM of MTX for 6 h. N ≥3. (C) Inhibition of Bj protein-elicited TNF-α release, in a 24-well culture dish format, stimulated with 0.5 µM of Bj protein for 6 h in the presence of 0–10 µM MTX. N ≥3. (D) Bj protein-elicited mitogenic activity for 10 h. Results are given in terms of relative cell growth. N ≥3. (E) Cell growth in the presence or absence of Bj protein (0.5 µM), or MTX (100 µM). N ≥3. (F) Time course of MTX cytotoxicity. RAW 264.7 cell growth was elucidated using the WST-8 cell proliferation assay and is shown as relative cell growth (%). Data represented as mean ± SE. *P<0.05, ****P<0.001.

To investigate the inhibitory effect of MTX on the HMGB1/RAGE interaction at the cellular level, we measured HMGB1-elicited TNF-α release *via* RAGE in RAW 264.7 cells using ELISA [22], [33], [34]. Specific RAGE stimulation with noncomplexed Bj protein (0.01–0.5 µM) for 6 h increased the release of TNF in a concentration-dependent manner in RAW 264.7 cells (Figure 5A). By contrast, MTX alone had no such effect during this period (Figure 5B). However, incubation of RAW 264.7 cells with 0.5 µM Bj protein in the presence of 1 or 10 µM MTX (i.e., 2-fold and 20-fold excess over Bj, respectively) resulted in a reproducible suppression of TNF release (Figure 5C). Together with the T7 phage display and SPR data, these findings support the idea that binding of MTX to K149-V175 in HMGB1 might interfere with the HMGB1/RAGE interaction, thereby showing the anti-inflammatory activity of MTX.

Besides TNF release, binding of HMGB1 to RAGE can also regulate cell motility and neurite outgrowth in RAW 264.7 cells [22], [35]. Thus, we further examined the effect of MTX for HMGB1-elicited mitogenic activity in RAW 264.7 cells. Treatment of the cells with 0.05–0.5 µM of Bj protein for 10 h facilitated mitogenic activity in a concentration-dependent manner (Figure 5D). When the cells were pre-incubated with 0.5 µM Bj protein in the presence of 100 µM MTX, Bj-stimulated mitogenic activity was significantly inhibited (Figure 5E). MTX (0.1–100 µM) alone was not toxic to RAW 264.7 cells after incubation for up to 10 h (Figure 5F). These results suggest that MTX might interfere with HMGB1-induced mitogenic activity. Thus, it seems likely that MTX inhibits HMGB1-induced cell locomotion by binding to K149-V175 in HMGB1 and preventing HMGB1/RAGE interaction.

## Discussion

Development of display cloning technology has significantly contributed to recent progress in the life sciences as well as other related studies. Here, we have described the use of T7 phage display to identify proteins that bind MTX, an anti-tumor drug with anti-inflammatory activity. Over the past decade, phage display has been applied to the determination of target proteins or peptides for small-molecule therapeutics [5]. Unlike using protein samples as bait, passive immobilization of drug-like small-molecules is not normally successful. Here, a biotinylated derivative was used to immobilize MTX onto a streptavidin-coated 96-well microplate. The length or position of the introduced biotin linker is a key factor in the synthetic design of the bait molecule. This is because the linker may render the biotinylated small molecule inaccessible to an internally located binding site on a protein, thereby preventing the comprehensive screening of potential binding candidates. On the other hand, development of the T7 phage system facilitates the display of larger proteins of up to 1200 amino acids on the C-terminus of the phage capsid protein. Indeed, even human proteins can be displayed by inserting a cDNA library into the cloning site of gene10 that encodes the capsid protein. This system is suitable for the screening of drug-binding proteins, including extracellularly-secreted cytokines or growth factors with a rapid turnover or low abundance. However, problems associated with folding, stability or size limitations will restrict the extent of the comprehensive screening. Indeed, no T7 phages that display DHFR or dCK, well-known molecular targets of MTX, were identified in this study [5]. The failure to detect these targets could have been due to the technical difficulties outlined above or from the choice of cDNA library and biopanning conditions used in our screening.

Nevertheless, using a mixture of two bio-MTX derivatives (Figure 1), we have identified T7 phages that display part of the HMGB1 protein (Figure 2B). HMGB1 is a multifunctional alarmin involved in inflammatory response and works as a transcriptional factor inside the nucleus and as a mediator of inflammation outside the cell [6]. Our experiments have revealed that, although the T7 phage-displayed HMGB1 fragment (K86-V175) contains Box B, TLR4-binding and RAGE-binding domains (Figure 3A), only RAGE-related events are significantly inhibited in the presence of MTX. This suggests that inhibition of HMGB1/RAGE interaction might be relevant to the anti-inflammatory effect of MTX. Indeed, on the T7 phage-displayed HMGB1 fragment (K86-V175), the RAGE-binding region was located on the C-terminus (Figure 3A) and was accessible to the immobilized MTX molecule, even with the shorter biotin linker. Furthermore, among the known HMGB1 receptors, RAGE is the only receptor that is stimulated by the unmodified and noncomplexed HMGB1 [9], [26]. The fact that binding of MTX to HMGB1 is independent of posttranscriptional modification or complex formation of HMGB1 correlates with our findings, because chimeric proteins displayed on T7 phage will be free from such modifications.

Previously, glycyrrhizin, a glycoconjugated triterpene that shows chemotactic and mitogenic activities, was reported as a bivalent ligand for HMGB1 [32]. The binding sites are located on Box A (F17, Q20, R23, E25, H26, K42, K43) and B (E107, R109, K113, G122, D123, A125, G129, N133) domains. However, the binding of glycyrrhizin to HMGB1 differs from that of MTX. Specifically, glycyrrhizin displays a weaker affinity constant (150 µM) than MTX as well as a moderate inhibitory effect for HMGB1-DNA interactions. Indeed, our experimental data indicates that, unlike glycyrrhizin, MTX specifically binds to the RAGE binding domain in HMGB1 (K149–K182) and inhibits the HMGB1/RAGE interaction. Moreover, MTX had no detectable effect on the interaction between HMGB1 and DNA. Docking simulation gave an unambiguous model for MTX association *via* N92 and other residues at the C terminus of helix III (D157, Y161, R162), which comprises a helix-loop-helix structure that resembles the first EF-hand of S100 proteins and functions as the RAGE binding domain [26], [36]. In NMR experiments to investigate the interaction of glycyrrhizin and HMGB1, no chemical-shift differences (CSD) were observed for the amino acid residues comprising helix III [32]. Thus, taken together, these data reveal important dissimilarities in the binding properties of HMGB1 with glycyrrhizin or MTX. Nonetheless, studies into the mode of action of both compounds clearly suggest that HMGB1 is a promising drug target for anti-inflammatory diseases. Further experiments will reveal the precise mode of docking between MTX and HMGB1.

Many studies have demonstrated the essential role of HMGB1 in acute and chronic inflammation such as RA [6]. Furthermore, the anti-inflammatory effects of MTX are undeniable given its success in clinical treatment [1]. Indeed, within the limited period of MTX incubation (∼10 h) in this study, we were able to detect the inhibition of a truncated HMGB1-elicited TNF-α release and mitogenic activities *via* RAGE. There are, however, technical difficulties associated with the long-term treatment of MTX, which displays significant cytotoxicity due to targeting other molecules such as DHFR or dCK. In addition, the complexity of HMGB1 biology makes the molecular biological studies challenging. A better understanding of the HMGB1/RAGE interaction will help define the anti-inflammatory mode of action of MTX.

According to the clinical guidelines, treatment of NSAIDs-ineffective chronic RA is initiated by oral administration of 10–15 mg of MTX per person per week, which allows reaching 1-hour serum level of MTX to be approximately 0.1–1 µM [37], [38], [39]. Wallace *et al.* reported that 1-hour serum level of ≥0.58 µM (0.3 mg/kg/week) are significantly associated with the therapeutic response in children with juvenile rheumatoid arthritis (JRA) [40]. Meanwhile, the dissociation constant (K_D_) between MTX and Bj obtained by SPR analysis was 0.24±0.01 µM (Table 1), which is very close to those concentrations. It must be remembered that various physiological conditions (*e.g.*, extent of acidic pH at the inflammation site) may influence the affinity strength between MTX and HMGB1 *in vivo*. Genetic factors, such as HMGB1/RAGE expression levels in each patient might also produce the individual differences regarding to the sensitivity to MTX. Further clinical investigations will clarify the causal relationship between MTX/HMGB1 interaction and therapeutic response by MTX in detail.

In conclusion, using the T7 phage display system, we have identified HMGB1 as a direct binding protein of MTX. Moreover, we have characterized the interaction between MTX and HMGB1. The binding of MTX to HMGB1 impedes HMGB1/RAGE interaction both *in vitro* and *in vivo*, thereby inhibiting the HMGB1-elicited TNF-α release and mitogenic activity. Our findings increase our understanding of the mode of action of MTX at the molecular level.

## Supporting Information

Figure S1 **SPR sensorgram with global fitting curve between bio-MTX and Al protein.** A solution containing various concentrations of Al (0.31–5 µM) was injected over the immobilized MTX-biotin on a SA sensor chip for 120 s and then dissociation was monitored for a further 120 s at a flow rate of 30 µl/min. Response curves were generated by subtraction of the background signals generated simultaneously on the control flow cell (bio-MTX-non-immobilized cell), and the injection of vehicle (0 µM Al).
(PDF)

Figure S2 **SPR sensorgram with global fitting curve between bio-MTX and Bj protein.** A Solution containing various concentrations of Bj (0.16–2.5 µM) was injected over the immobilized MTX-biotin on a SA sensor chip. Response curves were obtained using the same procedure as described for Al protein (refer to Figure S1).
(PDF)

Figure S3 **SPR sensorgram with fitting curve in associating process between bio-MTX and AlBj protein.** Five different concentrations of AlBj protein (0.63–10 µM) were injected over the immobilized MTX-biotin on a SA sensor chip. Responses were obtained as described in Figure S1.
(PDF)

Figure S4 **SPR sensorgram with global fitting curve between Bj protein and RAGE immobilized on a CM5 sensor chip in the absence of MTX.** Six different concentrations of Bj protein (0.31–10 µM) were injected over the immobilized RAGE and the response curves were obtained as described in Figure S1.
(PDF)

Figure S5 **SPR sensorgram with global fitting curve between Bj protein and RAGE immobilized on a CM5 sensor chip in the presence of MTX.** Six different concentrations of Bj protein (0.31–10 µM) with 1 mM MTX were injected over the immobilized RAGE and the response curves were obtained as described in Figure S1.
(PDF)

Table S1 **Kinetic parameters for the interaction between bio-MTX and Al protein.**
(PDF)

Table S2 **Kinetic parameters for the interaction between bio-MTX and Bj protein.**
(PDF)

Table S3 **SPR raw data for the interaction between bio-MTX and AlBj protein.**
(PDF)

Table S4 **Raw data from the SPR experiment to study the interaction between RAGE and Bj protein in the absence and presence of MTX (1 mM).**
(PDF)

## References

[1] Swierkot J, Szechinski J (2006) Methotrexate in rheumatoid arthritis. Pharmacol Rep 58: 473–492. 16963793

[2] Gargaro AR, Soteriou A, Frenkiel TA, Bauer CJ, Birdsall B, et al (1998) The solution structure of the complex of Lactobacillus casei dihydrofolate reductase with methotrexate. J Mol Biol 277: 119–134. 9514736 10.1006/jmbi.1997.1560

[3] Uga H, Kuramori C, Ohta A, Tsuboi Y, Tanaka H, et al (2006) A new mechanism of methotrexate action revealed by target screening with affinity beads. Mol Pharmacol 70: 1832–1839. 16936229 10.1124/mol.106.025866

[4] Majumdar S, Aggarwal BB (2001) Methotrexate suppresses NF-kappaB activation through inhibition of IkappaBalpha phosphorylation and degradation. J Immunol 167: 2911–2920. 11509639 10.4049/jimmunol.167.5.2911

[5] Takakusagi Y, Takakusagi K, Sugawara F, Sakaguchi K (2010) Use of phage display technology for the determination of the targets for small-molecule therapeutics. Expert Opin Drug Discov 5: 361–389. 22823088 10.1517/17460441003653155

[6] Harris HE, Andersson U, Pisetsky DS (2012) HMGB1: a multifunctional alarmin driving autoimmune and inflammatory disease. Nat Rev Rheumatol 8: 195–202. 22293756 10.1038/nrrheum.2011.222

[7] Ueda T, Yoshida M (2010) HMGB proteins and transcriptional regulation. Biochim Biophys Acta 1799: 114–118. 20123073 10.1016/j.bbagrm.2009.11.005

[8] Wang H, Bloom O, Zhang M, Vishnubhakat JM, Ombrellino M, et al (1999) HMG-1 as a late mediator of endotoxin lethality in mice. Science 285: 248–251. 10398600 10.1126/science.285.5425.248

[9] Hori O, Brett J, Slattery T, Cao R, Zhang J, et al (1995) The receptor for advanced glycation end products (RAGE) is a cellular binding site for amphoterin. Mediation of neurite outgrowth and co-expression of rage and amphoterin in the developing nervous system. J Biol Chem 270: 25752–25761. 7592757 10.1074/jbc.270.43.25752

[10] Fan J, Huennekens FM (1997) Biotin derivatives of folate compounds: synthesis and utilization for visualization and affinity purification of folate transport proteins. Methods Enzymol 281: 97–105. 9250972 10.1016/s0076-6879(97)81014-2

[11] Fan J, Vitols KS, Huennekens FM (1991) Biotin derivatives of methotrexate and folate. Synthesis and utilization for affinity purification of two membrane-associated folate transporters from L1210 cells. J Biol Chem 266: 14862–14865. 1869524

[12] Novagen (2009) OrientExpressTM cDNA Manual. Novagen TB247: 1109JN.

[13] Novagen (2009) T7 Select® System Manual. Novagen TB178: 1009JN.

[14] Takakusagi Y, Kuroiwa Y, Sugawara F, Sakaguchi K (2008) Identification of a methotrexate-binding peptide from a T7 phage display screen using a QCM device. Bioorg Med Chem 16: 7410–7414. 18602265 10.1016/j.bmc.2008.06.007

[15] Saito K, Kikuchi T, Shirakawa H, Yoshida M (1999) The stabilized structural array of two HMG1/2-boxes endowed by a linker sequence between them is requisite for the effective binding of HMG1 with DNA. J Biochem 125: 399–405. 9990140 10.1093/oxfordjournals.jbchem.a022300

[16] Takakusagi Y, Takakusagi K, Ida N, Takami M, Matsumoto Y, et al (2011) Binding region and interaction properties of sulfoquinovosylacylglycerol (SQAG) with human vascular endothelial growth factor 165 revealed by biosensor-based assays. Medchemcomm 2: 1188–1193.

[17] Zierler K (1989) Misuse of nonlinear Scatchard plots. Trends Biochem Sci 14: 314–317. 2799900 10.1016/0968-0004(89)90157-6

[18] Goutelle S, Maurin M, Rougier F, Barbaut X, Bourguignon L, et al (2008) The Hill equation: a review of its capabilities in pharmacological modelling. Fundam Clin Pharmacol 22: 633–648. 19049668 10.1111/j.1472-8206.2008.00633.x

[19] Hardman CH, Broadhurst RW, Raine AR, Grasser KD, Thomas JO, et al (1995) Structure of the A-domain of HMG1 and its interaction with DNA as studied by heteronuclear three- and four-dimensional NMR spectroscopy. Biochemistry 34: 16596–16607. 8527432 10.1021/bi00051a007

[20] Weir HM, Kraulis PJ, Hill CS, Raine AR, Laue ED, et al (1993) Structure of the HMG box motif in the B-domain of HMG1. EMBO J 12: 1311–1319. 8467791 10.1002/j.1460-2075.1993.tb05776.x PMC413342

[21] Stott K, Tang GS, Lee KB, Thomas JO (2006) Structure of a complex of tandem HMG boxes and DNA. J Mol Biol 360: 90–104. 16813837 10.1016/j.jmb.2006.04.059

[22] Yang H, Wang H, Czura CJ, Tracey KJ (2005) The cytokine activity of HMGB1. J Leukoc Biol 78: 1–8. 15734795 10.1189/jlb.1104648

[23] Taniguchi N, Kawahara K, Yone K, Hashiguchi T, Yamakuchi M, et al (2003) High mobility group box chromosomal protein 1 plays a role in the pathogenesis of rheumatoid arthritis as a novel cytokine. Arthritis Rheum 48: 971–981. 12687539 10.1002/art.10859

[24] Park JS, Svetkauskaite D, He Q, Kim JY, Strassheim D, et al (2004) Involvement of toll-like receptors 2 and 4 in cellular activation by high mobility group box 1 protein. J Biol Chem 279: 7370–7377. 14660645 10.1074/jbc.M306793200

[25] Li J, Kokkola R, Tabibzadeh S, Yang R, Ochani M, et al (2003) Structural basis for the proinflammatory cytokine activity of high mobility group box 1. Mol Med 9: 37–45. 12765338 PMC1430376

[26] Huttunen HJ, Fages C, Kuja-Panula J, Ridley AJ, Rauvala H (2002) Receptor for advanced glycation end products-binding COOH-terminal motif of amphoterin inhibits invasive migration and metastasis. Cancer Res 62: 4805–4811. 12183440

[27] Yamamoto A, Ando Y, Yoshioka K, Saito K, Tanabe T, et al (1997) Difference in affinity for DNA between HMG proteins 1 and 2 determined by surface plasmon resonance measurements. J Biochem 122: 586–594. 9348088 10.1093/oxfordjournals.jbchem.a021793

[28] Hreggvidsdottir HS, Lundberg AM, Aveberger AC, Klevenvall L, Andersson U, et al (2012) High mobility group box protein 1 (HMGB1)-partner molecule complexes enhance cytokine production by signaling through the partner molecule receptor. Mol Med 18: 224–230. 22076468 10.2119/molmed.2011.00327 PMC3320135

[29] Yang H, Hreggvidsdottir HS, Palmblad K, Wang H, Ochani M, et al (2010) A critical cysteine is required for HMGB1 binding to Toll-like receptor 4 and activation of macrophage cytokine release. Proc Natl Acad Sci U S A 107: 11942–11947. 20547845 10.1073/pnas.1003893107 PMC2900689

[30] Youn JH, Kwak MS, Wu J, Kim ES, Ji Y, et al (2011) Identification of lipopolysaccharide-binding peptide regions within HMGB1 and their effects on subclinical endotoxemia in a mouse model. Eur J Immunol 41: 2753–2762. 21660935 10.1002/eji.201141391 PMC3193378

[31] Schierbeck H, Wahamaa H, Andersson U, Harris HE (2010) Immunomodulatory drugs regulate HMGB1 release from activated human monocytes. Mol Med 16: 343–351. 20386869 10.2119/molmed.2010.00031 PMC2935946

[32] Mollica L, De Marchis F, Spitaleri A, Dallacosta C, Pennacchini D, et al (2007) Glycyrrhizin binds to high-mobility group box 1 protein and inhibits its cytokine activities. Chem Biol 14: 431–441. 17462578 10.1016/j.chembiol.2007.03.007

[33] Andersson U, Wang H, Palmblad K, Aveberger AC, Bloom O, et al (2000) High mobility group 1 protein (HMG-1) stimulates proinflammatory cytokine synthesis in human monocytes. J Exp Med 192: 565–570. 10952726 10.1084/jem.192.4.565 PMC2193240

[34] Kokkola R, Andersson A, Mullins G, Ostberg T, Treutiger CJ, et al (2005) RAGE is the major receptor for the proinflammatory activity of HMGB1 in rodent macrophages. Scand J Immunol 61: 1–9. 15644117 10.1111/j.0300-9475.2005.01534.x

[35] Palumbo R, Sampaolesi M, De Marchis F, Tonlorenzi R, Colombetti S, et al (2004) Extracellular HMGB1, a signal of tissue damage, induces mesoangioblast migration and proliferation. J Cell Biol 164: 441–449. 14744997 10.1083/jcb.200304135 PMC2172232

[36] Huttunen HJ, Rauvala H (2004) Amphoterin as an extracellular regulator of cell motility: from discovery to disease. J Intern Med 255: 351–366. 14871459 10.1111/j.1365-2796.2003.01301.x

[37] Smolen JS, Landewe R, Breedveld FC, Dougados M, Emery P, et al (2010) EULAR recommendations for the management of rheumatoid arthritis with synthetic and biological disease-modifying antirheumatic drugs. Ann Rheum Dis 69: 964–975. 20444750 10.1136/ard.2009.126532 PMC2935329

[38] Smolen JS, Aletaha D, Bijlsma JW, Breedveld FC, Boumpas D, et al (2010) Treating rheumatoid arthritis to target: recommendations of an international task force. Ann Rheum Dis 69: 631–637. 20215140 10.1136/ard.2009.123919 PMC3015099

[39] Visser K, Katchamart W, Loza E, Martinez-Lopez JA, Salliot C, et al (2009) Multinational evidence-based recommendations for the use of methotrexate in rheumatic disorders with a focus on rheumatoid arthritis: integrating systematic literature research and expert opinion of a broad international panel of rheumatologists in the 3E Initiative. Ann Rheum Dis 68: 1086–1093. 19033291 10.1136/ard.2008.094474 PMC2689523

[40] Wallace CA, Bleyer WA, Sherry DD, Salmonson KL, Wedgwood RJ (1989) Toxicity and serum levels of methotrexate in children with juvenile rheumatoid arthritis. Arthritis Rheum 32: 677–681. 2735961 10.1002/anr.1780320604

