# Supplementary figures and images for: Identification and Characterization of the Direct Interaction between Methotrexate (MTX) and High-Mobility Group Box 1 (HMGB1) Protein
Source: PLoS One. 2013 May 3;8(5):e63073. doi: 10.1371/journal.pone.0063073 (PMC3643934; doi:10.1371/journal.pone.0063073)

Figure S1

A

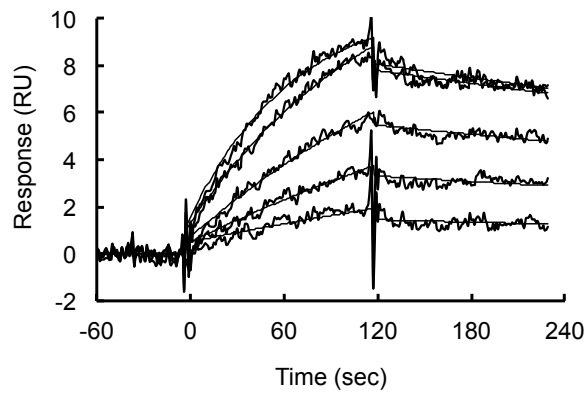

B

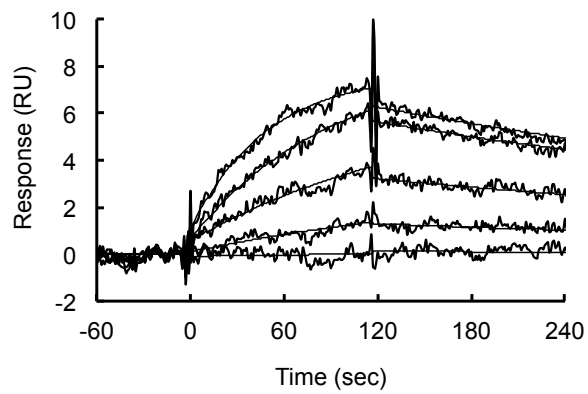

C

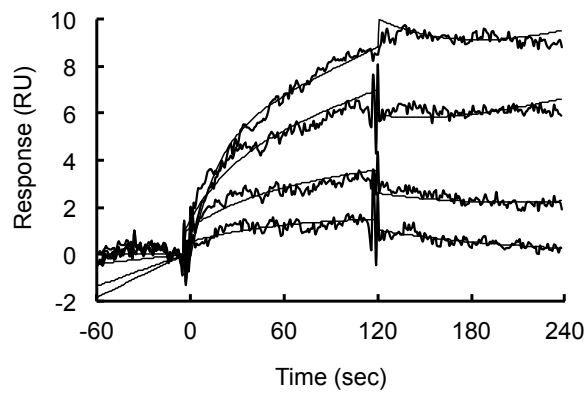

Supplement: Figure S1 — SPR sensorgram with global fitting curve between bio-MTX and Al protein. A solution containing various concentrations of Al (0.31–5 µM) was injected over the immobilized MTX-biotin on a SA sensor chip for 120 s and then dissociation was monitored for a further 120 s at a flow rate of 30 µl/min. Response curves were generated by subtraction of the background signals generated simultaneously on the control flow cell (bio-MTX-non-immobilized cell), and the injection of vehicle (0 µM Al). (PDF) [file pone.0063073.s001.pdf]

Figure S2

A

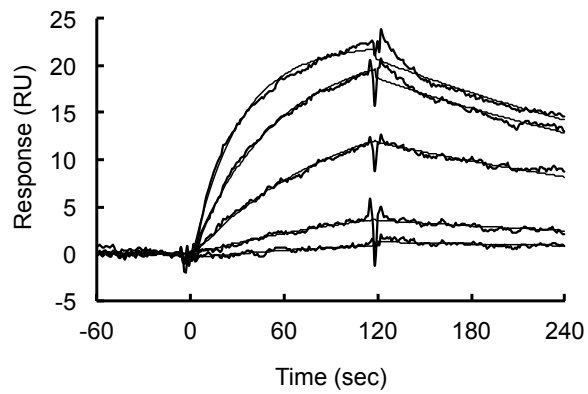

B

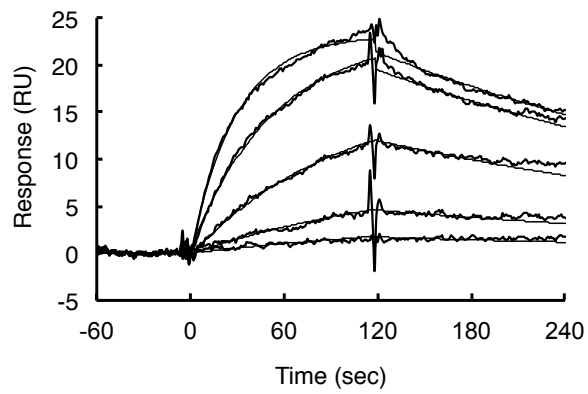

C

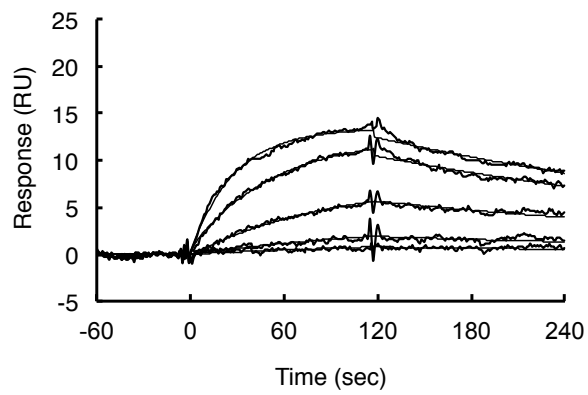

Supplement: Figure S2 — SPR sensorgram with global fitting curve between bio-MTX and Bj protein. A Solution containing various concentrations of Bj (0.16–2.5 µM) was injected over the immobilized MTX-biotin on a SA sensor chip. Response curves were obtained using the same procedure as described for Al protein (refer to Figure S1). (PDF) [file pone.0063073.s002.pdf]

Figure S3

A

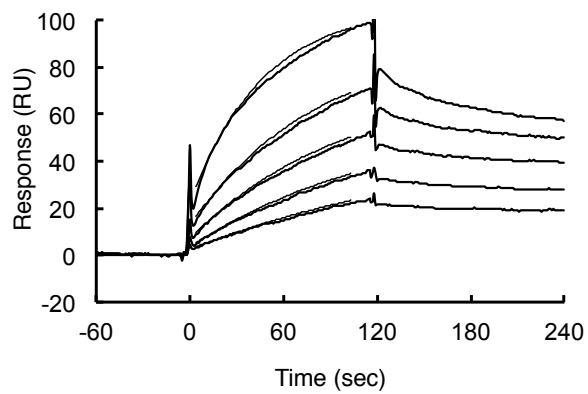

B

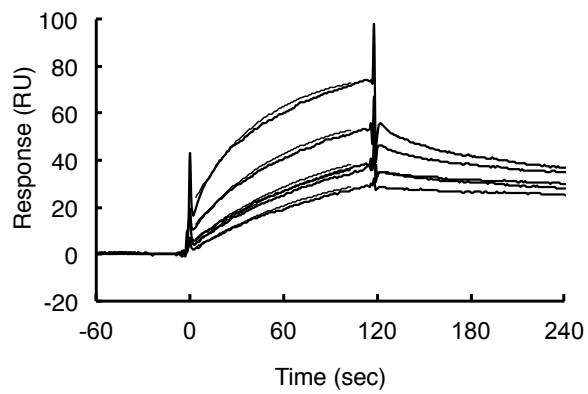

Supplement: Figure S3 — SPR sensorgram with fitting curve in associating process between bio-MTX and AlBj protein. Five different concentrations of AlBj protein (0.63–10 µM) were injected over the immobilized MTX-biotin on a SA sensor chip. Responses were obtained as described in Figure S1. (PDF) [file pone.0063073.s003.pdf]

Figure S4

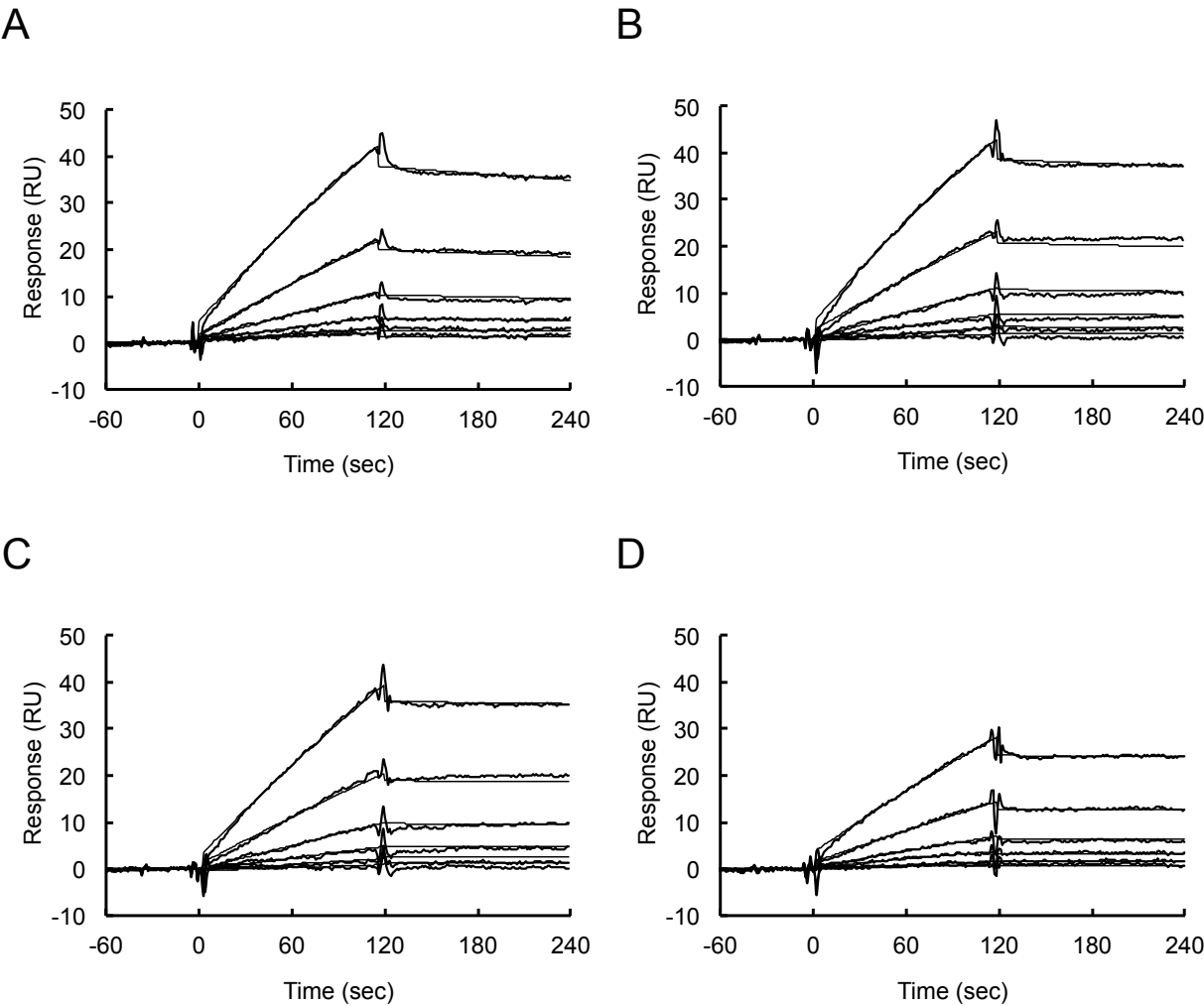

Supplement: Figure S4 — SPR sensorgram with global fitting curve between Bj protein and RAGE immobilized on a CM5 sensor chip in the absence of MTX. Six different concentrations of Bj protein (0.31–10 µM) were injected over the immobilized RAGE and the response curves were obtained as described in Figure S1. (PDF) [file pone.0063073.s004.pdf]

**Figure S5**

**A**

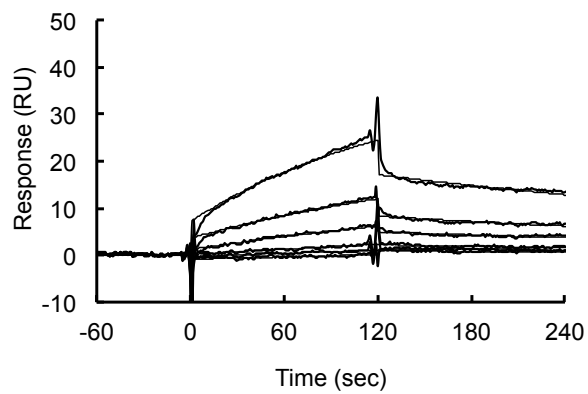

**B**

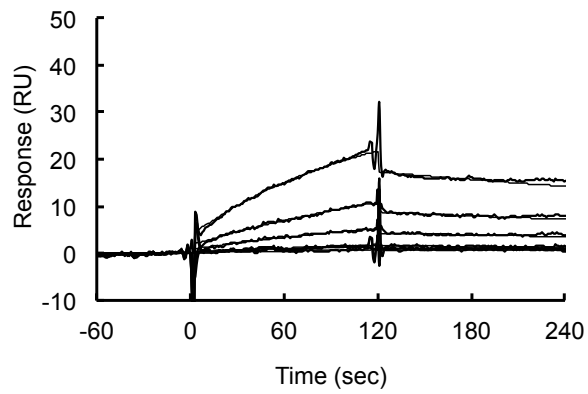

**C**

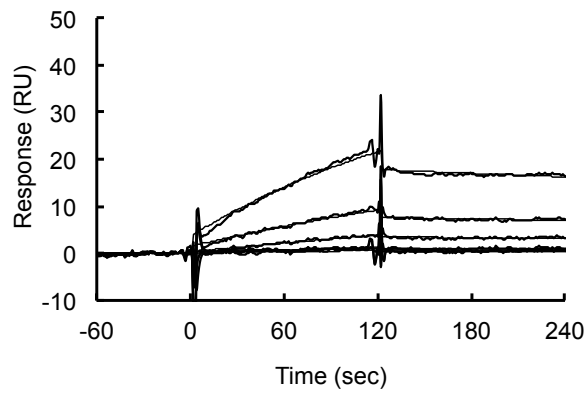

Supplement: Figure S5 — SPR sensorgram with global fitting curve between Bj protein and RAGE immobilized on a CM5 sensor chip in the presence of MTX. Six different concentrations of Bj protein (0.31–10 µM) with 1 mM MTX were injected over the immobilized RAGE and the response curves were obtained as described in Figure S1. (PDF) [file pone.0063073.s005.pdf]
