# Supplementary material for: Identification and Characterization of the Direct Interaction between Methotrexate (MTX) and High-Mobility Group Box 1 (HMGB1) Protein
Source: PLoS One. 2013 May 3;8(5):e63073. doi: 10.1371/journal.pone.0063073 (PMC3643934; doi:10.1371/journal.pone.0063073)
Supplement: Table S1 — Kinetic parameters for the interaction between bio-MTX and Al protein. (PDF) [file pone.0063073.s006.pdf]

Table S1

Entry	k_a ($\times10^3$ $M^{-1}s^{-1}$)	k_d ($\times10^{-3}$ s^{-1})	$K_D(k_d/k_a)$ (μM)	R_{max} (RU)	χ^2
A	3.69	1.95	0.53	6.5	0.042
B	2.78	1.21	0.44	15.4	0.049
C	19.5	10.5	0.54	4.6	0.075

Analytical conditions: PBS, 25 °C. k_a: association rate constant, k_d: dissociation rate constant, K_D: dissociation constant, R_{max}: maximum binding amount, χ^2: fitting to a 1:1 langmuir model using BIAevaluation 4.1 software (complete fitting; $\chi^2 = 0$).
