# Supplementary material for: Identification and Characterization of the Direct Interaction between Methotrexate (MTX) and High-Mobility Group Box 1 (HMGB1) Protein
Source: PLoS One. 2013 May 3;8(5):e63073. doi: 10.1371/journal.pone.0063073 (PMC3643934; doi:10.1371/journal.pone.0063073)
Supplement: Table S2 — Kinetic parameters for the interaction between bio-MTX and Bj protein. (PDF) [file pone.0063073.s007.pdf]

Table S2

Entry	k_a ($\times 10^3$ $M^{-1}s^{-1}$)	k_d ($\times 10^{-3}$ s^{-1})	$K_D(k_d/k_a)$ (μM)	R_{max} (RU)	χ^2
A	12.1	3.10	0.26	18.5	0.118
B	12.6	3.07	0.24	19.6	0.093
C	13.0	3.02	0.23	9.7	0.071
