# Supplementary material for: Identification and Characterization of the Direct Interaction between Methotrexate (MTX) and High-Mobility Group Box 1 (HMGB1) Protein
Source: PLoS One. 2013 May 3;8(5):e63073. doi: 10.1371/journal.pone.0063073 (PMC3643934; doi:10.1371/journal.pone.0063073)
Supplement: Table S3 — SPR raw data for the interaction between bio-MTX and AlBj protein. (PDF) [file pone.0063073.s008.pdf]

Table S3

Conc. of AlBj (μM)	R_{eq} (RU) A (χ^2 = 0.306)	B (χ^2 = 0.215)	Average
0.625	46.3	39.5	42.9
1.25	54.8	44.3	49.6
2.5	64.5	47.1	55.8
5	75.2	50.3	62.8
10	76.8	55.1	66.0

R_{eq}: Response at equilibrium between immobilized bio-MTX and AlBj protein
