# Supplementary material for: Identification and Characterization of the Direct Interaction between Methotrexate (MTX) and High-Mobility Group Box 1 (HMGB1) Protein
Source: PLoS One. 2013 May 3;8(5):e63073. doi: 10.1371/journal.pone.0063073 (PMC3643934; doi:10.1371/journal.pone.0063073)
Supplement: Table S4 — Raw data from the SPR experiment to study the interaction between RAGE and Bj protein in the absence and presence of MTX (1 mM). (PDF) [file pone.0063073.s009.pdf]

Table S4

Bj/RAGE/MTX (−)	R_{eq} (RU)				
Conc. of Bj (μM)	A (χ^2 = 0.173)	B (χ^2 = 0.508)	C (χ^2 = 0.45)	D (χ^2 = 0.092)	Average
0	0	0	0	0	0
0.3125	16.8	28.7	59.6	50.9	39
0.625	30.8	48.3	89.3	74.7	60.8
1.25	52.9	73.4	119	97.8	85.8
2.5	82.7	99.1	143	116	110.2
5	115	120	159	127	130.3
10	143	134	168	134	144.8

Bj/RAGE/MTX (+)	R_{eq} (RU)			
Conc. of Bj (μM)	A (χ^2 = 0.101)	B (χ^2 = 0.055)	C (χ^2 = 0.098)	Average
0	0	0	0	0
0.3125	3.5	3.9	4.9	4.1
0.625	5.2	5.2	5.9	5.4
1.25	7.2	6.2	7.3	6.9
2.5	14.2	13.2	16.9	14.8
5	19	19.4	24.8	19.2
10	29.6	27.1	37.2	28.4

R_{eq}: Response at equilibrium between immobilized RAGE and Bj protein
